# Nuclear Factor 90, a cellular dsRNA binding protein inhibits the HIV Rev-export function

**DOI:** 10.1186/1742-4690-3-83

**Published:** 2006-11-24

**Authors:** Silvio Urcuqui-Inchima, Maria Eugenia Castaño, Danièle Hernandez-Verdun, Georges St-Laurent, Ajit Kumar

**Affiliations:** 1 Grupo de Inmunovirología, Corporación Biogénesis, Universidad de Antioquia, A.A. 1226, Medellín, Colombia; 2 Institut Jacques Monod, CNRS, University Paris VI and Paris VII, 2 place Jussieu, 75251 Paris Cedex 05, France; 3 Department of Biochemistry and Molecular Biology, The George Washington University, Washington, D.C. 20037, USA

## Abstract

**Background:**

The HIV Rev protein is known to facilitate export of incompletely spliced and unspliced viral transcripts to the cytoplasm, a necessary step in virus life cycle. The Rev-mediated nucleo-cytoplasmic transport of nascent viral transcripts, dependents on interaction of Rev with the RRE RNA structural element present in the target RNAs. The C-terminal variant of dsRNA-binding nuclear protein 90 (NF90ctv) has been shown to markedly attenuate viral replication in stably transduced HIV-1 target cell line. Here we examined a mechanism of interference of viral life cycle involving Rev-NF90ctv interaction.

**Results:**

Since Rev:RRE complex formations depend on protein:RNA and protein:protein interactions, we investigated whether the expression of NF90ctv might interfere with Rev-mediated export of RRE-containing transcripts. When HeLa cells expressed both NF90ctv and Rev protein, we observed that NF90ctv inhibited the Rev-mediated RNA transport. In particular, three regions of NF90ctv protein are involved in blocking Rev function. Moreover, interaction of NF90ctv with the RRE RNA resulted in the expression of a reporter protein coding sequences linked to the RRE structure. Moreover, Rev influenced the subcellular localization of NF90ctv, and this process is leptomycin B sensitive.

**Conclusion:**

The dsRNA binding protein, NF90ctv competes with HIV Rev function at two levels, by competitive protein:protein interaction involving Rev binding to specific domains of NF90ctv, as well as by its binding to the RRE-RNA structure. Our results are consistent with a model of Rev-mediated HIV-1 RNA export that envisions Rev-multimerization, a process interrupted by NF90ctv.

## Background

Upon entering an uninfected host cell, the single-stranded RNA genome of Human immunodeficiency virus type 1 (HIV-1) is copied into DNA by the viral reverse transcriptase. Following integration of DNA into the host genome, transcriptional activation of the proviral DNA generates progeny virions. The post-integration events of transcription, RNA splicing, transport and translation of viral mRNAs are regulated by coordinate interaction with host proteins [1]. Strict dependence of viral gene expression on host factors particularly those with protein:RNA and protein:protein binding properties, are useful targets to explore novel antiviral therapy.

Regulation of HIV-1 gene expression is controlled mainly by the two virus encoded proteins, Tat responsible for processive transcription elongation, and Rev which regulates transport of unspliced and incompletely spliced viral transcripts from the nucleus to the cytoplasm. These two regulatory proteins function by binding to structured RNA domains present in the viral transcripts, the trans-activation responsive RNA (TAR) and the Rev response element (RRE) respectively.

The functional domains of Rev include an N-terminal nuclear localization signal (NLS) rich in Arg-residues, and a C-terminal nuclear export signal (NES) rich in Leu-residues. The NES of Rev interacts with host proteins that are critical for RNA export, and the NLS binds to the RRE-RNA structure, and is also involved in Rev-homodimerization [2-4]. After dissociation from RRE in the cytoplasm, the NLS of Rev binds to importin-β (Imp-β), allowing its import back into the nucleus. Once in the nucleus, Rev interacts with the RRE RNA present in the incompletely spliced and unspliced viral transcripts. The newly formed Rev:RRE complex recruits proteins such as Crm1 [3] or eIF5A [2] that are essential cofactors in regulating nuclear export [5,6]. Interaction of Rev with Crm1 occurs via the NES present in both proteins [7] a process inhibited by the leptomycin B (LMB). The NES domains play a critical role in the intracellular localization of viral and cellular proteins [8-10].

The Rev:RRE:Crm1 complex is translocated through the nuclear pore complex to the cytoplasm. This translocation is dependant on the RNA helicase activity of DDX3, which binds to the Rev:RRE:Crm1 complex on the nuclear side of the Nuclear Pore Complex and accompanies it through to the cytoplasmic side [11,12]. After dissociation, the viral transcripts are recognized by the translation machinery for synthesis of viral structural proteins [2].

In murine fibroblast A9 cells, export of HIV-1 transcripts mediated by Rev is restricted due to as yet unidentified host cell factor(s) that block Rev-mediated transport. However the Rex protein, an analogue of Rev encoded by the Human T cell leukemia virus type 1 (HTLV-1) is able to mediate RNA transport in murine cells. Marques et al. [13] reported that a chimeric protein containing the N-terminal region of Rev (amino acids 1–79) comprising the NLS, and the C-terminal region of Rex (amino acids 79–95) comprising the NES region of Rex, restored Rev-mediated RNA transport in A9 cells, suggesting that a putative murine cell inhibitor of Rev-function might recognize NES domain of Rev.

NF90 is encoded by the *ilf-3 *gene, which by alternative splicing [14,15] gives rise to a family of double stranded RNA binding domain(DRBD) proteins that include NF90, NF110, MPP_4 _90, MPP_4 _110, ILF-3, NFAR-1 and NFAR-2, which are implicated in different functions such as transcription, RNA splicing, RNA editing and export. It is unclear if any of the DRBD proteins directly influence Rev-dependent RNA transport.

**Figure 1:**
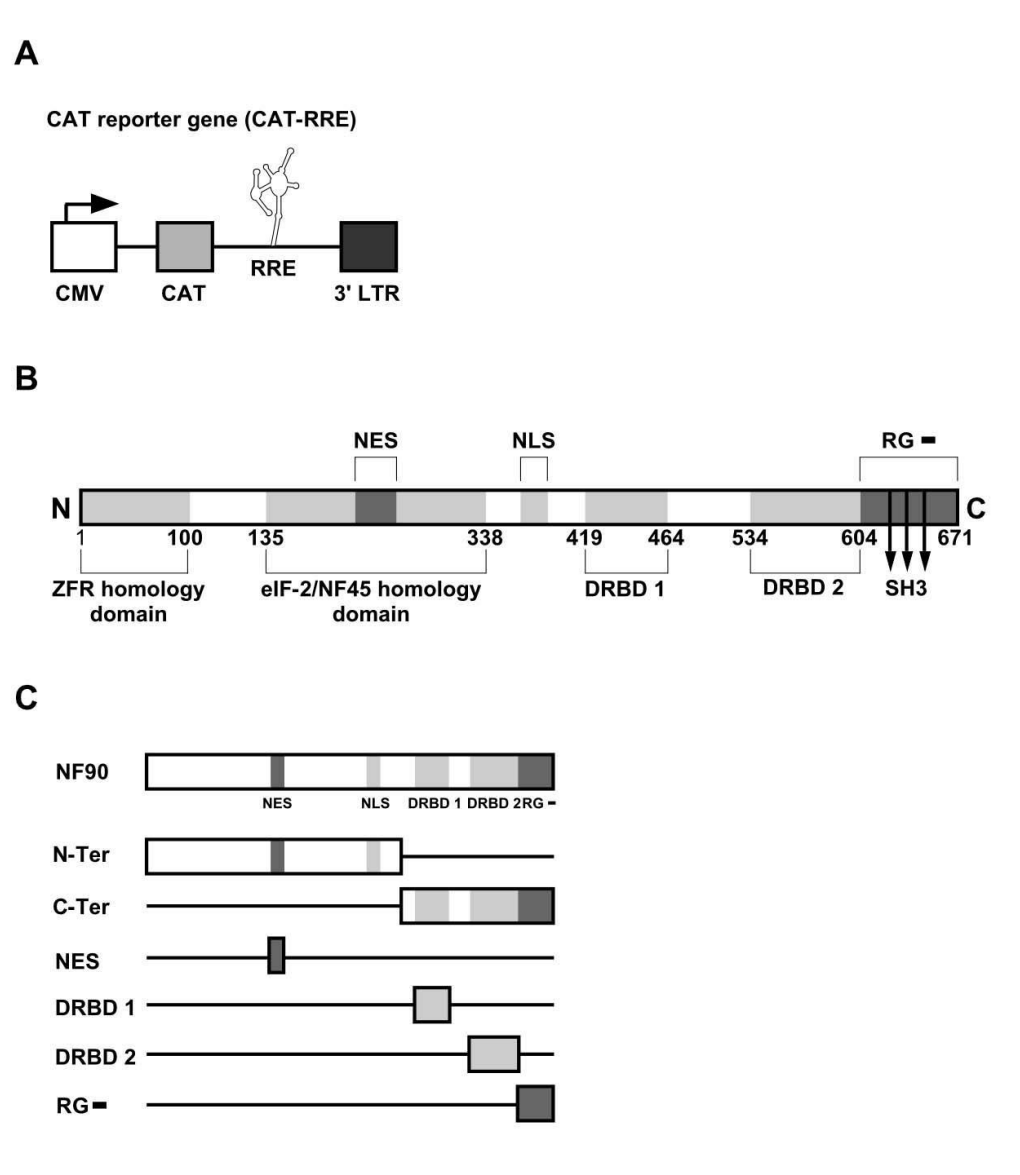
Schematic representation of (A) pCMV128 (CAT-RRE reporter), and (B) the NF90ctv regions investigated here. (C) The NF90ctv domains were cloned into pCI-neo and used in this study. The vertical arrows correspond to the SH3 motifs present in RG- region.

The proteins in this family are characterized by the presence of two C-terminal dsRNA binding domains (DRBD 1 and DRBD 2; Fig. 1) that allow the protein to bind structured and duplexed RNAs [16-18]. NF90 has been found associated with different viral RNAs, such as human Adeno-associated virus RNA_II_, the encapsidation signal ε in Hepatitis B virus RNA, and bovine viral diarrhea virus RNA [18-20]. Its central NLS domain and its N-terminal sequence homologous to zinc finger regions interact with DNA and RNA [21], and a Leu-rich region functions as an NES [19]. The NES region is similar to the NES consensus sequence described in HIV-1 Rev.

We studied a unique form of NF90, designated NF90ctv [22]. The C-terminal variant, NF90ctv is distinct from other proteins encoded by *ilf-3 *in that its C-terminal 67 amino acids (Fig. 1) is Arg-Gly-poor (RG-), whereas the corresponding C-terminal region is Arg-Gly-rich (RG+) in all the other proteins of this family. NF90 is predominantly a nuclear protein. In interphase cells however, about half of the NF90 is tethered in the nucleus by RNA bound to protein with dsRNA-dinding motif, while in mitosis NF90 is phosphorylated and translocated to the cytoplasm, without loss of its affinity for dsRNA [23]. It has been reported that NF90 in the cytoplasm can stabilize the IL-2 mRNA by binding to its AU-rich elements [24]. Moreover, its presence in complexes with the 3' untranslated region (UTR) of Tau mRNA [25] indicates that NF90 is part of the protein complex that interacts with the Tau mRNA, and is essential for its exonal translocation. Thus the NF90 family of proteins, due largely to their dsRNA recognition function, may affect gene expression by targeted localization of structured RNA molecules.

Among the proteins encoded by the *ilf-3 *gene, NF90/NFAR's proteins have been reported to serve as substrate for PKR [26]. NF90 protein has characteristics analogous to those of interferon (IFN) type 1 [22] such as the capacity to activate genes involved in defense against viral infection. NF90 was originally reported to be a transcription factor, although it does not possess DNA binding domains [16]. NF90 can negatively or positively regulate expression of a reporter gene, depending on the viral promoter used [27]. The report that NF90ctv arrests HIV-1 replication in human osteosarcoma-derived cells [22], supports the argument that ectopic expression of NF90ctv results in overall stimulation of IFN response genes.

We reason that the attenuation of HIV-1 replication in NF90ctv expressing cells may in part be due to the affinity of NF90ctv protein to the structured RNA molecules essential in viral life cycle. That is, in addition to generalized induction of the innate antiviral response pathway in NF90ctv-expressing cells [22], the binding properties of NF90ctv to structured RNAs are likely to interfere with specific steps of viral life cycle, particularly those that rely on precise protein:RNA interactions. Thus, the overall antiviral response of NF90ctv may result from the stimulated innate immunity of the host following virus infection as well as from the ability of NF90ctv to interfere with specific steps in the viral life cycle. Here we examined the protein:protein and protein-dsRNA interaction properties of NF90ctv that inhibits HIV-1 replication by blocking Rev function. The results suggest that NF90ctv negatively affects the export of HIV-1 transcripts regulated by Rev. The NF90ctv-mediated inhibition of Rev involved three specific regions of NF90ctv protein.

## Results

### NF90ctv inhibits Rev-dependent CAT activity

**Figure 2:**
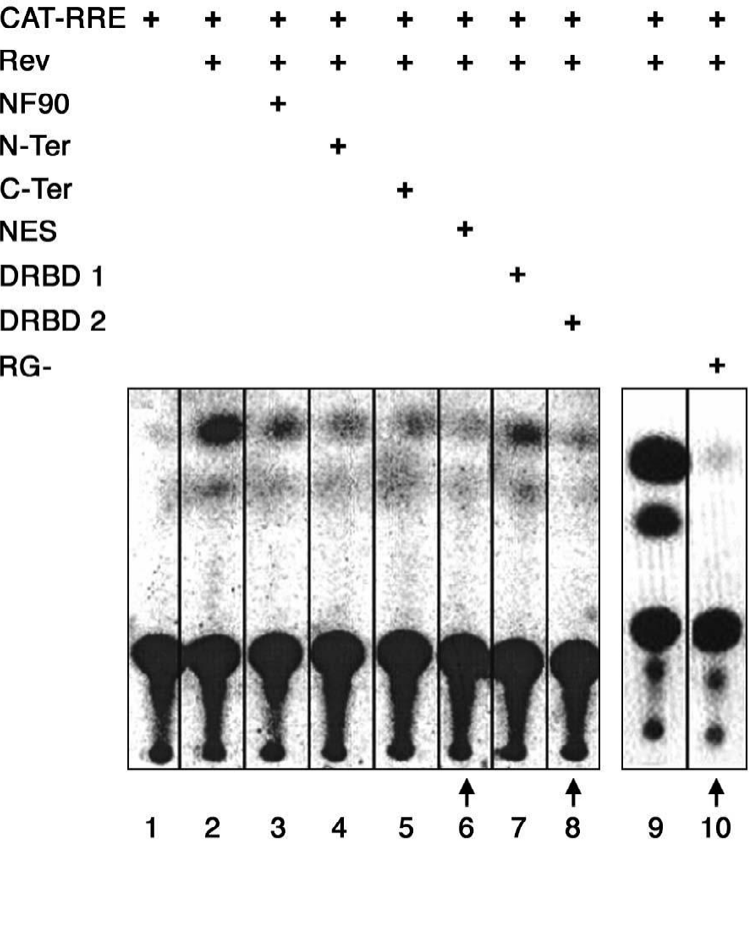
Effect of NF90ctv protein domains on the level of expression of CAT. HeLa cells were cotransfected with CAT-RRE, and with different constructs that code for Rev protein and the NF90ctv domains. After cell lysis, ^14^C-labelled chloramphenicol and acetyl CoA were added to the extracts and the CAT activity determined. The construct included in each assay is indicated above the lanes. Vertical arrows highlight the constructs leading to the strongest decrease in acetylation of chloramphenicol. Similar results were obtained in three independent experiments. When the cells were transfected only with the empty expression vector for NF90 (pCI-neo), acetylation of chloramphenicol was not observed (not shown).

The CAT-RRE reporter gene (pCMV128; Fig. 1A) was used to evaluate the export of transcripts that depend on Rev-RRE interaction. In this reporter system the cytoplasmic expression of the CAT coding sequence depends on the presence of functional Rev which allows efficient export of unspliced and incompletely spliced mRNAs. Rev function was determined by the level of acetylated chloramphenicol expression in HeLa cell extracts. As shown in Figure 2, CAT activity was absent when the cells were transfected with the reporter gene alone (lane 1), or with the pCI-neo empty expression vector (not shown). In the presence of Rev (pRSV/Rev), there resulted a high level of CAT activity (lane 2). However, in cells expressing NF90ctv along with Rev, the CAT activity was greatly reduced (compare lanes 2 and 3) indicating that NF90ctv interfered with the RNA export activity of Rev.

Next we characterized specific region(s) of NF90ctv (Fig. 1B) implicated in inhibition of Rev function. The 5'- or the 3'-regions of the NF90ctv coding sequence were cloned separately into the pCI-neo vector and the resulting constructs were used to transfect HeLa cells in the presence of the reporter pCMV128 and the Rev expression vector, pRSV/Rev. Both the N-terminal and the C-terminal regions of NF90ctv inhibited Rev function (Fig. 2, lanes 4 and 5, respectively), suggesting that at least two regions of NF90ctv contain sequences that affect the Rev function.

As outlined (Fig. 1B), two dsRNA binding motifs (DRBD 1 and DRBD 2) are located in the C-terminal half of NF90ctv, where as the N-terminal region of NF90ctv may be involved in protein-protein interactions. By comparison with other proteins in the NF90 family, the N-terminal half of NF90ctv contains an NES region (Fig. 1B), and its C-terminal 67 amino acid sequence is deficient in arginine/glycine residues (RG-). To evaluate the influence of specific NF90ctv domains on Rev-dependent CAT activity, the NES, DRBD 1, DRBD 2 and RG- coding regions were cloned into the pCI-neo vector (Fig. 1C), and used in cotransfection experiments in the presence of CAT-RRE reporter, pCMV128 and the Rev expression vector, pRSV/Rev. We first verified that the NES, DRBD 2 and RG- fragments without NLS, translocate to the nucleus. Indeed, when these NF90 protein domains were cloned into the fluorescent tagged vector, pmRFP and expressed as fusion proteins, they were observed in the cytoplasm as well as in the nucleus (data not shown).

As shown in Figure 2, the NES, DRBD 2 and RG- regions inhibited the Rev dependent CAT activity (lanes 6–8 and 10 respectively); DRBD 1 effect was less pronounced (lane 10); however, RG- showed a higher inhibitory effect on Rev-mediated RNA export. These results suggest that three regions of NF90ctv, the NES, DRBD 2 and RG-, are involved in blocking Rev function, and collectively they would strongly interfere with Rev function, possibly involving protein:protein or protein:RNA interactions. Results similar to those presented in Figure 2 were obtained with three independent transfection assays with each of the NF90ctv domains. In control experiments, using the empty pCI-neo vector, there was no induction of the CAT reporter gene with or without Rev (data not shown).

### NF90 interact with REV protein

The N-terminus of NF90ctv protein includes a region that is likely to be involved in protein:protein interactions [26]. In addition, its C-terminal RG- domain includes three SH3 motifs (PXXP) that are known to be important in certain protein:protein interactions [28,29]. Hence a possible mechanism of NF90ctv-mediated inhibition of Rev function may include sequestration of Rev involving NF90ctv:Rev protein interaction. We tested the possibility by co-expressing Rev and NF90ctv in HeLa cells transfected with pRSV/Rev or pOZ-Flag/NF90. We assessed the expression of two proteins by Western blotting, using α-Rev_31–50 _and anti-FLAG antibodies. Results showed that both Rev and NF90ctv proteins are expressed in HeLa cells (data not shown).

**Figure 3:**
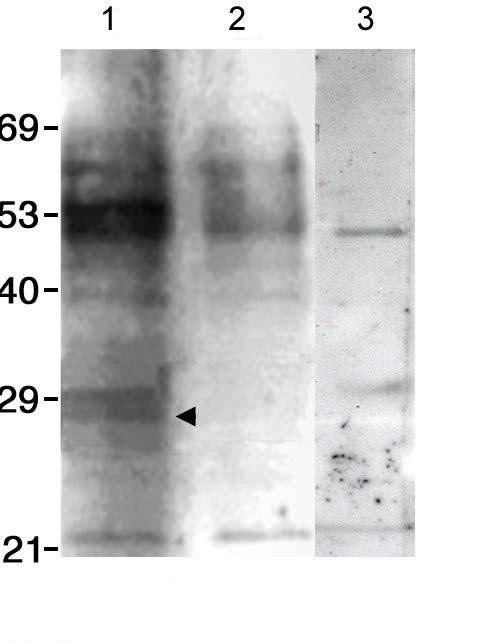
Immunoprecipitation. HeLa cells were cotransfected with pRSV/Rev and pOZ-Flag/NF90; 24 h post-transfection, the cells were lysed, α-Flag antibodies were added and the proteins precipitated by adding protein A agarose. The samples were analyzed by SDS-PAGE, and the proteins transferred to a PVDF membrane were immunodetected using α-Rev_31–50 _antibodies. Lane 1, extracts from HeLa cells cotransfected with both constructs. Lane 2, extracts from untransfected HeLa cells. Lane 3, same amount of HeLa cell extract prior to immunoprecipitation (input control). Arrow indicates the protein band (~28 kDa) corresponding to Rev.

**Figure 4:**
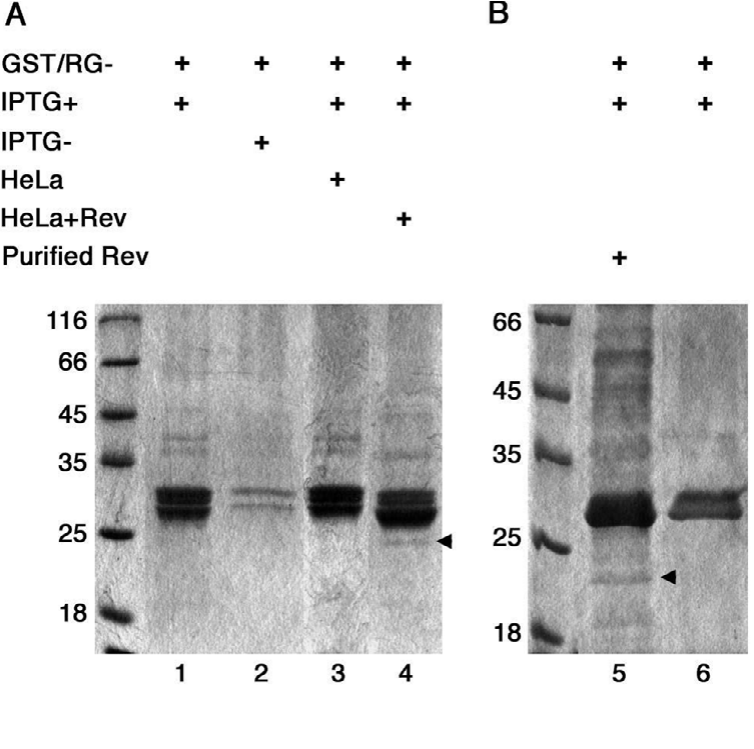
Interaction between the NF90ctv RG- domain and Rev by affinity chromatography. (A) The GST/RG- recombinant protein was expressed in *E. coli*, the cell extracts coupled to a glutathione-Sepharose 4B column was used in pull-down assays with HeLa cell extracts previously transfected with pRSV/Rev (lane 4) or the control lysate (lane 3). A protein band corresponding to Rev (arrowhead, lane 4) was detected when extracts from HeLa cells that expressed Rev were added to the GST/RG- protein bound column; such a band was absent from control HeLa cell extract (lane 3). Lane 1, GST/RG- purified from *E. coli *induced by IPTG, and lane 2, purified from non induced *E. coli *cells. (B) Similar assays performed with purified Rev protein from *E. coli *in place of HeLa cells extracts. Arrow in lane 5 indicates the position of Rev. This band was not observed in absence of Rev (lane 6).

To examine NF90ctv:Rev interaction we immunoprecipitated HeLa cell lysates with α-Flag antibodies, 24 hr after cotransfection with pRSV/Rev and pOZ-Flag/NF90. The resulting protein complex was enriched by addition of Protein A-agarose beads, resolved by SDS-PAGE and analyzed by Western blot using the α-Rev_31–50 _antibodies. The results show that Rev co-immunoprecipitated with α-Flag antibodies (Fig. 3, lane 1) in cells that expressed both the Rev and Flag/NF90 proteins, yielding a band of 28 kDa. In contrast, Rev was not detected in extracts of HeLa cells that were not transfected with the Rev-expressing plasmid, pRSV/Rev (lane 2), or in control assays where cell extracts were used prior to immunoprecipitation (input cell extracts; lane 3). The results suggest that Rev specifically associates with NF90ctv *in vivo*. As control a 28 kDa band was observed with the purified Rev protein (Fig. 4B, lane 5). Taken together, the results suggest that interaction between NF90ctv and Rev proteins could inhibit Rev function either by sequestration of Rev in NF90ctv:Rev protein complexes that would lead to a decrease in available Rev protein for Rev-mediated RRE interaction. Alternatively, interaction of NF90ctv with another cellular protein(s) may be involved in blocking Rev export activity.

### The RG- region of NF90ctv is involved in interaction with Rev

The C-terminal 67 amino acids (604–671) of NF90ctv designated RG-, possess three SH3 motifs, that appear to be involved in protein:protein interactions [30]. This region of NF90ctv was cloned into pGEX-4T-3 leading to pGST/RG- that is expected to yield a recombinant GST/RG- protein of ~33 kDa. The protein expressed in *E. coli *was purified by affinity chromatography and analyzed by Western blotting using either a polyclonal α-GST/RG- or α-GST antibodies. With both types of antibodies, a protein band migrating at the level of the recombinant GST/RG- protein was detected. No ~33 kDa band appeared in similar Western blots with preimmune sera (data not shown).

The purified GST/RG- protein was used to study the interaction of the RG- domain of NF90ctv with Rev. It was coupled to a glutathione-Sepharose 4B column, and used in pull-down assays by adding extracts from HeLa cells that express Rev, or with purified Rev protein. After washing to remove unbound proteins, possible Rev:RG- complexes were eluted, resolved by SDS-PAGE and visualized by Coomassie blue staining. As shown in Figure 4 lane 4, when extract from HeLa cells expressing Rev was added to the glutathione-Sepharose column, an additional band of ~25 kDa corresponding to Rev protein was detected. This band was absent from control samples, *i.e. *lysates from IPTG-induced *E. coli *cells (lane 1), or from uninduced (lane 2) *E. coli *cells or with extracts from untransfected HeLa cells (lane 3). Similar results were obtained when purified Rev expressed in *E. coli *replaced the HeLa cell extracts previously transfected with pRSV/Rev (lane 5). It should be noted that in all cases a double band of about 33 kDa was observed for GST/RG- but only the faster-migrating band was recognized by the α-GST/RG- or α-GST (Amersham) antibodies. The results indicate that the RG- region of NF90ctv is involved in direct interaction with Rev, in agreement with the results obtained by immunoprecipitation of full length NF90ctv (Fig. 3, lane 1).

### NF90ctv and Rev show similar subcellular localization

**Figure 5:**
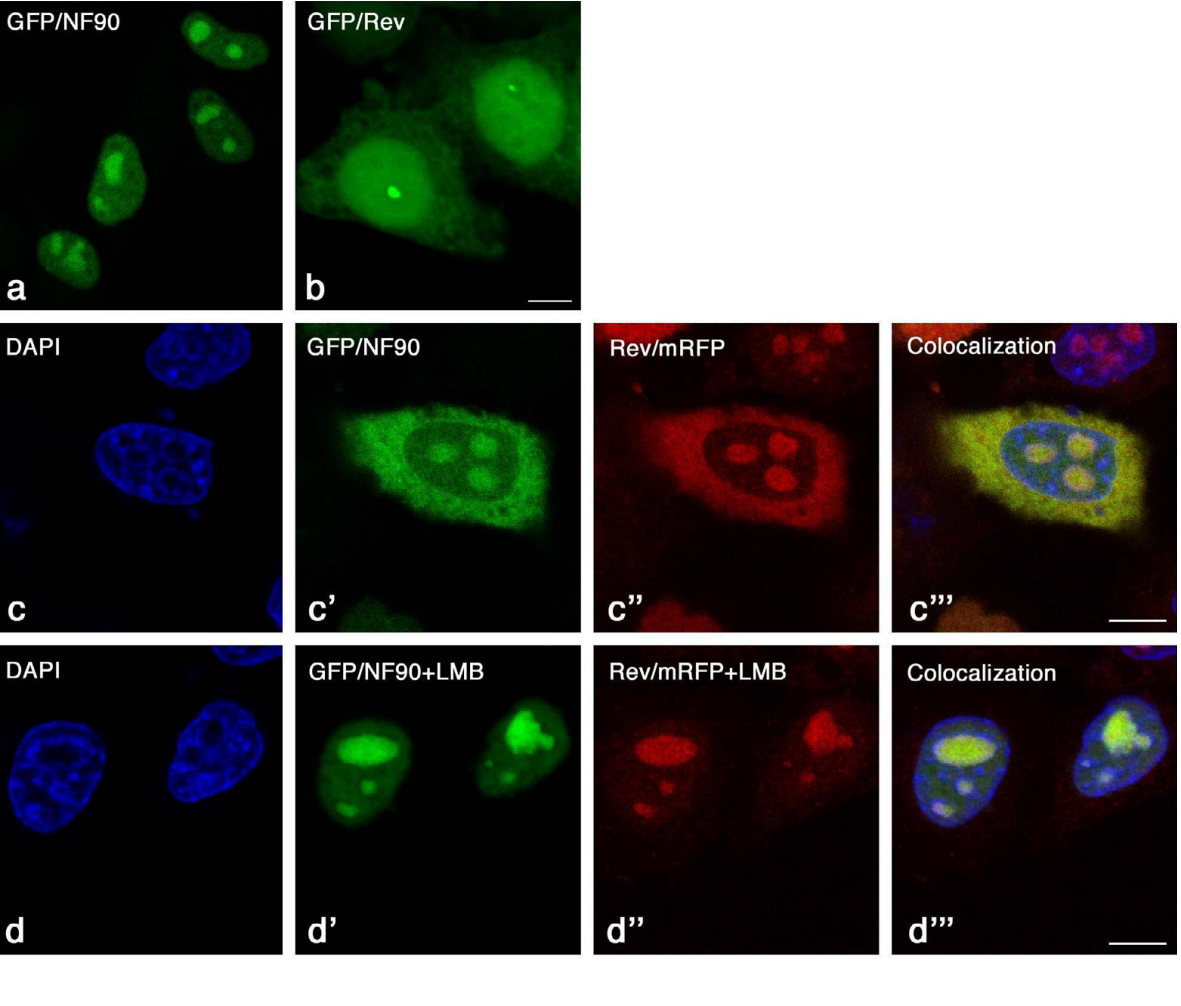
Subcellular localization of the Rev and NF90ctv proteins observed by fluorescence microscopy in confocal optical sections. HeLa cells were transfected with pGFP/NF90 or pGFP/Rev and GFP was visualized with an Argon laser at 488 nm. NF90 localized in the nucleus and nucleolus; Rev localized in the nucleus, nucleolus and cytoplasm (panels *a *and *b*). To study the effect of NF90ctv on Rev localization, HeLa cells were cotransfected with both expression vectors and the proteins were observed in confocal optical sections; the mRFP was visualized using a Krypton laser at 568 nm. Both protein colocalized in the similar subcellular compartments (*c'-c''; d'-d'' *are the same as *c'-c'''*). In the presence of 50 ng/ml of LMB and both proteins localized mostly in the nucleolus. Bar = 5 μm.

To investigate whether expression of NF90ctv affects the cellular localization of Rev, we investigated the localization of NF90ctv-GFP or of Rev-mRFP in Hela cells 24 h after transfection with either pNF90ctv/GFP or pGFP/Rev vectors alone, or after co-transfection with pGFP/NF90 + pRev/mRFP (Fig. 5c). In agreement with previous observations [13], Rev protein alone localized in the nucleus, mostly in the nucleolus as confirmed by DAPI staining, and to a lesser extent in the cytoplasm (Fig. 5b). Similar results were observed when HeLa cells were transfected with pRev/mRFP (data not shown). NF90ctv alone localized mostly in the nucleus and in the nucleolus (Fig. 5a). Cells co-transfected with Rev and NF90ctv showed co-localization of the two proteins in the same cellular compartments (Fig. 5c'''), i.e. in the nucleolus and the cytoplasm; in this case NF90ctv localized mostly in the nucleolus and cytoplasm, and to lesser extent in the nucleus. The results suggest that the subcellular distribution of NF90ctv is altered in the presence of Rev: both proteins localized in the nucleus and in the cytoplasm.

Interestingly, in the case of HeLa cells co-transfected with pGFP/NF90 + pRev/mRFP in the presence of LMB, Rev was concentrated entirely in the nucleolus, whereas NF90 was present both in the nucleus and in the nucleolus (Fig. 5d'–d'''), mostly in the nucleolus.

### NF90ctv influences the expression of gene linked to RRE structure

**Figure 6:**
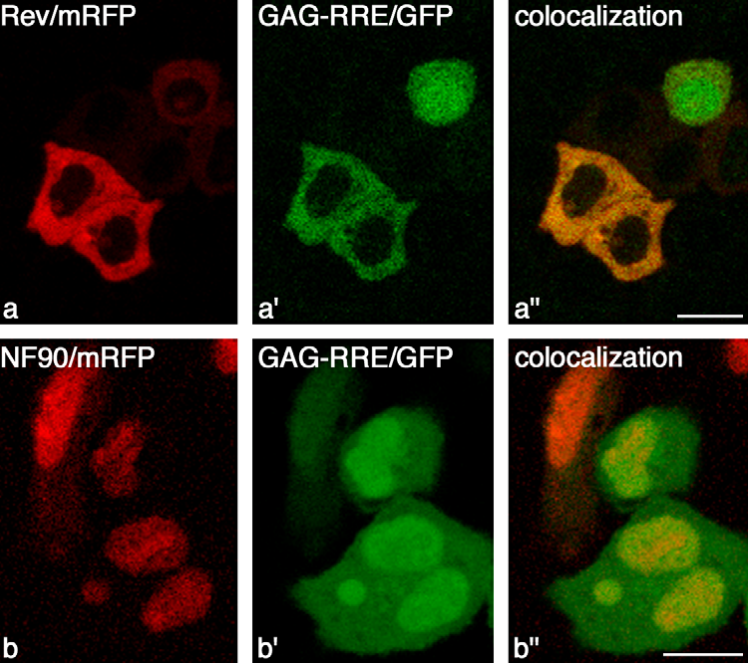
NF90ctv affects structured-RNA translocation. HeLa cells were cotransfected with pRev/mRFP and pSGT-5(SDM/RRE/CM-GFP) or with pNF90/mRFP and pSGT-5(SDM/RRE/CM-GFP). The constructs represent HIV-1 Gag and GFP genes linked to RRE, making their expression dependent on Rev. The reporter gene (GFP) linked to RRE is expressed either in presence of Rev or NF90ctv (*a *and *b*, respectively). As controls HeLa cells were transfected with each construct alone. In presence the RRE, Rev localized mainly in the cytoplasm (*a*), colocalized with the GFP reporter (*a''*). However, NF90ctv in the presence of RRE, preferentially localized in the nucleus/nucleoli (*b*), and GFP reporter was distributed in the cytoplasm and in the nucleus.

To determine whether NF90ctv is directly implicated in export (and translation) of transcripts linked to RRE, we used a construct containing the *Gag *and *GFP *coding sequences linked to RRE. In this reporter system as well, the cytoplasmic translocation and translation of Gag-GFP protein is Rev-dependent. Furthermore, our results showed that Rev/GFP retained nuclear/nucleolar localization (Fig. 5b), as did NF90ctv which localized in the nuclear/nucleolar compartment (Fig 5a). As shown (Fig. 6), Gag-*GFP *mRNA that is linked to RRE, is expressed, transported to the cytoplasm and translated either in presence of Rev or the NF90ctv (Fig. 6a' and 6b' respectively). Rev protein in the presence of RRE, is transported to the cytoplasm (Fig 6a), where it is tracked with GFP (Fig. 6a"). In presence of NF90ctv (Fig. 6b), Gag-GFP is distributed throughout the cell (Fig 6b'), however, NF90ctv is mostly localized in the nucleus/nucleolus (Fig. 6b''), suggesting that RRE influences localization of NF90ctv. Overall, the results show that either Rev protein (Rev/mRFP) or NF90ctv (NF90/mRFP) influence Gag-RRE-GFP expression, suggesting that NF90ctv may influence the nucleocytoplasmic translocation of RRE containing transcripts, analogous to Rev function. The results shown in figure 6 were observed in 4 independent experiments.

## Discussion

The space of interactions between viral and host elements is complex, and our understanding of these dynamics is incomplete. In this study we explored whether the NF90ctv-mediated inhibition of HIV-1 Rev involved Rev-NF90ctv interaction. The results presented here show that NF90ctv inhibits the Rev mediated export of viral RNA. To refine this observation, and study its molecular mechanisms, we created a series of vectors containing segments of the NF90ctv gene to use in transfection studies. We investigated the possibility that specific regions of NF90ctv may be involved in blocking Rev-mediated RNA export activity, by expressing the NF90ctv regions in HeLa cells. Three regions of NF90ctv in particular diminished Rev function, the NES, DRBD 2 and the C-terminal RG-.

The region of NF90ctv with the greatest inhibition of Rev function, the RG- domain, consists of the C-terminal 67 amino acids. This domain contains three SH3 motifs that are rich in Pro, which has been described to be involved in protein:protein interactions [31]. SH3 motifs are highly conserved and are present in many proteins that interact with RNAs. We suggest that the SH3 motifs of NF90ctv may be involved in the Rev:NF90ctv interaction to block Rev function.

In addition, studies have indicated that NF90 possesses several regions capable of protein:protein interactions; including PKR [32,33], NF45 [16], and eIF-2 [26]. Protein motifs capable of interacting with dsRNAs can also participate in protein:protein interactions, such as homodimerization, as in the case of Rev. The active homodimers of Rev protein form complexes with the RRE structure [34,35]. Proteins with dsRNA binding motifs can also interact with dsRNA ligands bound to proteins implicated in innate immunity and defence against viral infections, such as PKR [36,37]. The detailed elucidation these interactions will be required to understand the dynamics of viral – host interactions.

**Figure 7:**
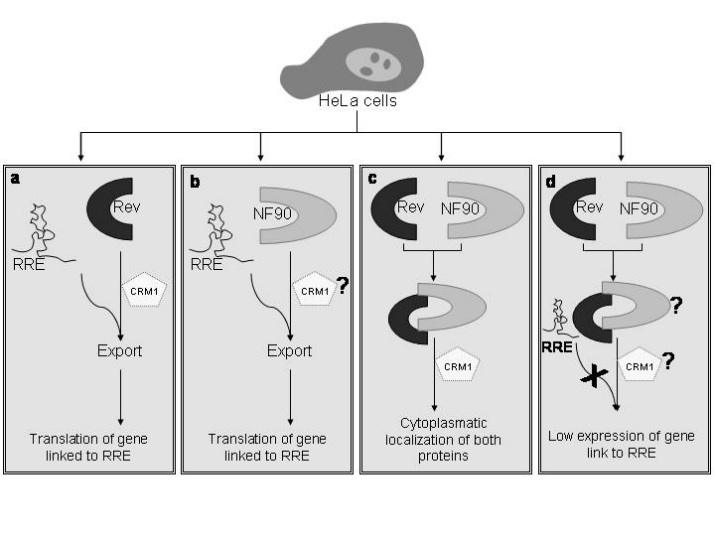
Model for regulation of Rev protein by NF90. The HeLa cells are cotransfected with the respective plasmids. In (a), the transcript of the gene linked to RRE is recognized by Rev and forms a complex with CRM1, it is exported to the cytoplasm, where it is translated by the ribosome. In (b) based on the data presented herein, we suggest that NF90 is able to recognize the transcript of the gene linked to RRE, and then it is exported to the cytoplasm and translated. In (c) we suggest that this process is CRM1-dependent. Because our immunoprecipitation results show that NF90 interacts with Rev and their colocalization in the cytoplasm is CRM1-dependent, In (d) we propose that the affinity of Rev or NF90 for RRE, is affected by protein-protein interaction (NF90-Rev), with as a consequence low expression of the gene linked to RRE; another possibility would be that the interaction of NF90 with Rev, alters the conformation of Rev, allowing decreased access to RRE.

The NES region of NF90ctv used in this study comprises amino acids 81–190 that include a Leu-rich sequence, present in the different NF90 isoforms encoded by the *ifl*-3 gene [14]. This region contains the LX_3_LX_3_LXL sequence, similar to the consensus LX_2–3_LX_2–3_LXL reported to be part of the NES in Rev [38]. The NES domain of NF90ctv has been suggested to play an important role in the shuttling of the protein between the nucleus and the cytoplasm that is required for IL-2 mRNA translocation and stabilization [24]. Both Rev export [7] and NF90ctv export are LMB-sensitive. Since the NES motif located in the N-terminal region of NF90ctv is similar to the hydrophobic Leu-rich region present in proteins [24] known to recruit the exportin Crm1 [3], we suggest that export of NF90ctv to the cytoplasm may be Crm1-dependent (Fig. 7b). This is supported by the observation that in cells treated with leptomycin B (Fig. 5d) NF90ctv is restricted in the nucleus (mostly in the nucleolus), suggesting that Crm1 recognizes the NES domain of NF90ctv. Following cell activation with 12-O-tetradecanoyl phorbol-beta-acetate and calcium ionophore A23187, a major portion of GFP/NF90 was translocated to the cytoplasm, and this translocation was inhibited by pretreatment with LMB. When NF90ctv is overexpressed in presence of the Gag gene linked to RRE, the Gag-RRE transcript is translocated to the cytoplasm (Fig. 6). We propose that competition between NF90ctv and Rev for Crm1 binding is a possible mechanism of interference with Rev function and that the interaction between NF90ctv and Rev influences the affinity of viral protein for RRE RNA structure (Fig. 7d).

However, since NF90ctv contains two dsRNA binding motifs, a direct interaction between NF90ctv and the HIV-1 RRE RNA structure may be equally important in blocking Rev function. These two DRBDs bind to the small and highly structured human Adenovirus transcript designated Adeno-associated virus RNA [18], to the highly conserved encapsidation signal ε of the Hepatitis B pregenomic RNA [19], and also to the 3' UTR of Tau mRNA [25]. We investigated whether NF90ctv could bind the RRE structure. Our results suggest that NF90ctv is able to bind to RRE, leading to the expression of the protein coding sequence linked to the RRE-RNA structure. Our results suggest that interaction between NF90ctv and Rev protein (Fig 7c), as well as direct interaction between NF90ctv and RRE RNA structure may affect RNA export from the nucleus (Fig. 7b). We suggest that competition between NF90ctv and Rev for RRE binding could in part explain the decrease of Gag expression. However, the overall molecular mechanisms of NF90ctv mediated antiviral response will require further studies.

Rev protein has been reported to reside mainly in the nucleus [2], and is translocated to cytoplasm in presence of RRE (Fig. 6a and Fig. 7a). Rev shuttles between the nucleus and cytoplasm, since it harbours the NLS domain that interacts with Importin β. The NES domain of Rev is recognized by Crm1/exportin 1 [39]. Substitution of Rev and RRE by an export promoting sequence construct, RTE m26 – CTE, produces infectious virus [40], suggesting that export is indeed a primary function of Rev:RRE. Similar to Rev, NF90 has been described mainly in the nucleus yet, it can shuttle between the nucleus and the cytoplasm due to its NES and NLS motifs [24]. When NF90ctv is expressed in presence of RRE RNA structure (GFP-RRE), it is tethered in the nucleus, whereas mRNA linked to RRE would normally be exported to the cytoplasm.

Overall, the results are consistent with a mechanism of inhibition of Rev based on the interaction of Rev protein with specific domains of NF90ctv. The Rev-NF90ctv interaction may prevent Rev homodimer formation, an essential step in interaction of Rev with RRE. For the export of unspliced or incompletely spliced transcripts to be efficient, Rev must be imported back into the nucleus where it must reach a specific concentration [2] to induce complex formation with the RRE. The transdominant Rev mutant, RevM10, that inhibits Rev activity [41], is restricted to the nucleus and competes with Rev for interaction with the RRE. Rev-dependent export has been reported to be specifically inhibited by LMB [42] which prevents the interaction between Rev and Crm1. Likewise Sam68ΔC, (i.e. the cell protein Sam68 devoid of its C-terminal domain), inhibits Rev activity, thereby preventing the transcripts from entering the translation machinery [43]. The authors conclude that Sam68ΔC leads to perinuclear accumulation of the unspliced or incompletely spliced mRNAs of HIV-1.

The results described here suggest a novel means of inhibition of HIV-1 Rev function based on direct interaction between NF90ctv and Rev, interfering with the export activity of Rev (Fig. 7). To our knowledge, no cellular protein has to date been reported to have such an effect. The antiretroviral compounds presently in use may be of limited use due to emerging drug resistant viral strains. Results described here open the possibility of exploring novel therapeutic approaches against HIV-1/AIDS, based on NF90-mediated inhibition of Rev.

## Methods

### Constructs and plasmids

The pEFX90 vector harboring the entire human NF90 coding sequence was kindly provided by P. N. Kao (Stanford Univ., Calif.). To produce Flag/NF90, the NF90 coding sequence was recovered from pEFX90 by *Nco*I digestion and subcloned into the pUC18 vector upstream of and in-frame with the Flag-coding sequence. The Flag/NF90 coding sequence was then excised by *Nsi*I and *Msc*I digestion and ligated into the pOZ vector kindly provided by Dr. B. Howard (NIH) and previously cleaved by *Xho*I and *Nsi*I, yielding the pOZ-Flag/NF90.

The pCI-neo/NF90 construct that allows expression of NF90ctv was described previously [22]. The NF90N-terminal and NF90C-terminal coding sequences were recovered from pCI-neo/NF90 by *Nhe*I/*Xba*I and *Xba*I/*Not*I digestion respectively and cloned into pCI-neo vector cleaved with the same enzymes, producing pCI-neo/N-ter NF90ctv and pCI-neo/C-ter NF90ctv respectively. To express other regions of NF90ctv proteins (NES, DRBD 1, DRBD 2, RG-; Fig. 1C) the appropriate regions of cDNA were amplified from pCI-neo/NF90 by PCR using specific primers (Table 1) and after *EcoR*I/*Sma*I digestion the products were inserted into pCI-neo using the same restriction enzymes.

**Table 1:** Primers used to amplify regions of NF90ctv by PCR for cloning into the pCI-neo vector.

	Amplified region	Construct
5'-primer: 5'GGGGGGGAATTCGCTAGCCGCCACCAATGCAGAAGACGGAGCACATGAC3' 3'-primer: 5'GGGGGGCCCGGGGCGGCCGACTAGATGGTCAGGGACAATGGACGCTCTTT3'	NES	pCI-neo/NES NF90
5'-primer: 5'GGGGGGGAATTCGCTAGCCGCCACCAATGAATGCCCTGATGCGGTTGAA3' 3'-primer: 5'GGGGGGCCCGGGGCGGCCGACTAAGCACCCGTCGGCAAGCCCATGTCCTG3'	DRBD 1	pCI-neo/DRBD 1 NF90
5'-primer: 5'GGGGGGGAATTCGCTAGCCGCCACCAATGAAGAACCCAGTCATGGAGCTG3' 3-primer: 5'GGGGGGCCCGGGGCGGCCGACTAAGGGAAAAGTTTTTCTAGGGCAGCAAG3'	DRBD 2	pCI-neo/DRBD 2 NF90
5'-primer: 5'GGGGGGGTCGACCGCCACCAATGAAGCTTTTCCCTGACACCCCTCTCGCC3' 3'-primer: 5'GGGGGGCCCGGGCGGCCGCCTATGTAGCCTCCATGGTTGGCGCCACCAAA3'	RG-	pCI-neo/RG- NF90

The pcsRev/GFP was kindly provided by Dr. George Pavlakis (National Cancer Institute, Frederick, Md). The reporter plasmid pCMV128 (provided by Dr. Thomas J. Hope, Northwestern University, Chicago) carries the second intron of HIV-1 with the RRE structure downstream of the CAT coding sequence (Fig. 1A) and therefore the expression of CAT strictly depends on Rev:RRE interaction for export of the CAT coding sequence [13]. pRSV/Rev that expresses Rev was kindly provided by Dr. T. J. Hope; the pSGT-5(SDM/RRE/CM-GFP) was kindly provided by Dr. S. Arya (NCI/NIH) and contains the HIV-1 Gag gene and the GFP gene linked to HIV RRE structure. The GFP-coding pEGFP-N1 was from Clontech.

To obtain the pRev/mRFP construct that expresses the monomeric red fluorescent protein (mRFP), the following strategy was used. The mRFP cassette was amplified from pcDNA3 (kindly provided by R. Y. Tsien, University of California, San Diego) using the 5'primer GGATCCGCGGCAGACCATGGCTAGCA and the 3'primer GCGGCCGCTTAGGCGCCGGTGGAGTG. The 5'primer presents a *BamH*I site (underlined) and a Kozak sequence, and the 3'primer a *Not*I site (underlined). On the other hand, pEGF-N1, which expresses the GFP, was cleaved with *BamH*I and *Not*I to delete the EGFP cassette, and replaced it by the mRFP PCR product to obtain the pmRFP construct used to transform *Escherichia coli *DH5α. Rev was amplified from pRSV/Rev using the 5'primer GAATTCTGCCGCCACCATGGCAGGAAGAAGCGGA (*EcoR*I underlined) and the 3'primer CCCGGGTTCTTTAGCTCCTGACTCCAA (*Sma*I underlined). The PCR product was digested with *EcoR*I/*Sma*I and ligated into pmRFP previously digested with the same enzymes to obtain the pRev/mRFP construct.

To introduce the RG- region of NF90ctv (Fig. 1C) into pGEX-4T-3 (Amershan), the corresponding region was released from pCI-neo/RG- NF90 using *EcoR*I/*Sma*I and cloned into pGEX-4T-3, yielding pGST/RG- that codes for the GST/RG- protein.

### Cell culture and transfection

A human osteosarcoma cell line stably transduced with CD4 and CXCR4, a T-tropic HIV-1 target cell line (GHOST/CD4+/CXCR4+), was maintained as described [22] except that fetal bovine serum was from Invitrogen, or from Vecol, Colombia. Cells were plated at ~3 × 10^5 ^cells per 35 mm-diameter dish 24 h before transfection with 2 μg each of the appropriate plasmid DNA using Superfect (Qiagen) following the manufacturer's instructions. Cells were harvested 24 or 48 h after transfection and lysed as described [13]. The protein concentration of lysates was determined by the Bradford assay (Bio-Rad).

To evaluate expression of Rev and Flag/NF90 following co-transfection with pRSV/Rev and pOZ-Flag/NF90, cell extracts were analyzed by SDS-PAGE, the proteins transferred to a polyvinylidene-difluoride membrane (PVDF; Bio-Rad), and immunodetected with α-Rev_31–50 _or α-Rev_71–90 _antibodies (generously supplied by S. Marques, George Washington University), or α-Flag antibodies (Sigma).

### CAT assay

HeLa cells were co-transfected with the plasmid constructs outlined in Table 1. To normalize transfection efficiency, the pEGFP-N1 control plasmid was included in each transfection assay. Cell lysates containing equal amounts of protein were used in the CAT assays performed as previously described [13]. The results are presented as fold increase or decrease in CAT activity measured as chloramphenicol acetylation normalized proteins from cells transfected with the pCMV128 reporter plasmid alone or the pCI-neo empty expression vector for NF90 as negative control.

### Immunoprecipitation and immunoblot analysis

For the immunoprecipitation assays, 4 × 10^5 ^HeLa cells were grown to about 80% confluence and cotransfected with 1.5 μg each of pRSV/Rev and pOZ-Flag/NF90, and after 24 h extracts were prepared as described above. Cell extracts (35 μl) were preincubated overnight at 4°C with 5 μl of α-Flag antibodies with gentle shaking. Protein A agarose (10 μl; Invitrogen) was then added and incubation continued for 3 h. The precipitate was recovered by centrifugation, washed 3 times in PBS 1× and the resin suspended in 20 μl of 4 × Laemmli buffer. The protein suspension was analyzed by SDS-PAGE, transferred to a PVDF membrane and probed using α-Rev_31–50 _antibodies. The same amount of HeLa cell extract prior to immunoprecipitation was used as input control in western blot assays.

### Expression and purification of the recombinant GST/RG- protein in *E. coli*

*E. coli *XL1 Blue cells transformed with pGST/RG- were induced or not overnight with 0.5 mM IPTG and lysed using lysis buffer (50 mM Tris-Cl pH 8.0, 1 mM EDTA, 100 mM NaCl and 1 mg/ml of lysozyme). The recombinant protein was purified using a glutathione-Sepharose 4B column (MicroSpin™ GST Purification Module, Amersham) following the manufacturer's instructions. The recombinant protein was verified by SDS-PAGE and by immunoblotting with α-GST (Amersham) or α-GST/RG- polyclonal antibodies.

### Determination of NF90 RG-/Rev interaction by affinity chromatography

After binding the GST/RG- protein to the glutathione-Sepharose 4B column, extracts from 10^6 ^HeLa cells transfected with pRSV/Rev, or 15 μg of purified Rev protein [44] [obtained through the AIDS Research and Reference Reagent Program, Division of AIDS, and NIAID, NIH: HIV-1 (wild type) from D. Rekosh, M.-L. Hammarskjöld, and M. Orsini] were added to the column. After several washes to remove unbound protein, the GST/RG-:Rev protein complex was eluted and analyzed by SDS-PAGE and the proteins detected by Coomassie staining.

### Cell localization

To determine if NF90ctv influences the cellular localization of Rev, HeLa cells (8 × 10^4^) were co-transfected either with 1 μ pRev/mRFP alone, with 1 μ pRev/mRFP + 1 μ pGFP/NF90 or with 1 μ pGFP/NF90 alone; 1 μ pmRFP was used as negative control. After 24 h, the cells were fixed for 20 min with 2% paraformaldehyde at room temperature. Following three washes with 1 × PBS, the nuclei were stained with 4-6-diamidino-2-phenylindol (DAPI), the cells were mounted with Citifluor (Canterbury, UK) and the GFP or mRFP was detected using a Leica SP2 AOBS confocal microscope with a 63×, 1.32NA PlanApo lens using an Argon laser (488 nm) or a Krypton laser (568 nm), respectively. The images were assembled using Adobe PhotoShop.

To study the effect of leptomycin B (LMB) on the subcellular localization of Rev and NF90ctv, HeLa cells were co-transfected as described above, and 24 h later, 50 ng/ml of LMB were added and incubation continued for 2 h at 37°C in 5% CO_2_. The cells were then fixed following the procedure described above and observed using a Leica SP2 AOBS confocal microscope.

### RRE-binding study

To examine if NF90 is able to bind the RRE RNA, the HeLa cells were transfected with either 1 μ pRev/mRFP alone or with 0.4 μ pSGT-5(SDM/RRE/CM-GFP) or with 1 μ pNF90/mRFP with or without 0.4 μ pSGT-5(SDM/RRE/CM-GFP). As negative control, we used the HeLa cells trasnfected only with pSGT-5(SDM/RRE/CM-GFP). After 24 h, the GFP or mRFP was detected by confocal microscopy, as described above.

## Competing interests

The author(s) declare that they have no competing interests.

## Authors' contributions

MEC carried out the studies, participated in the design and conception of the project. DH-V participated in the design and conception of the project and helped draft the manuscript. AK and GSL participated in the design and conception of the project and helped draft the manuscript. SU-I carried out the studies, participated in the design and conception of the project and drafted the manuscript. All authors read and approved the final manuscript.

## References

[B1] Cochrane AW, McNally MT, Mouland AJ (2006). The Retrovirus RNA Trafficking granule: from birth to maturity. Retrovirology.

[B2] Kjems J, Askjaer P (2000). Rev protein and its cellular partners. Adv Pharmacol.

[B3] Fornerod M, Ohno M, Yoshida M, Mattaj IW (1997). CRM1 is an export receptor for leucine-rich nuclear export signals. Cell.

[B4] Pollard VW, Malim MH (1998). The HIV-1 Rev protein. Annu Rev Microbiol.

[B5] Fukuda M, Asano S, Nakamura T, Adachi M, Yoshida M, Yanagida M, Nishida E (1997). CRM1 is responsible for intracellular transport mediated by the nuclear export signal. Nature.

[B6] Neville M, Stutz F, Lee L, Davis LI, Rosbash M (1997). The importin-beta family member Crm1p bridges the interaction between Rev and the nuclear pore complex during nuclear export. Curr Biol.

[B7] Askjaer P, Jensen TH, Nilsson J, Englmeier L, Kjems J (1998). The specificity of the CRM1-Rev nuclear export signal interaction is mediated by RanGTP. J Biol Chem.

[B8] Gorlich D, Kutay U (1999). Transport between the cell nucleus and the cytoplasm. Annu Rev Cell Dev Biol.

[B9] Mattaj IW, Englmeier L (1998). Nucleocytoplasmic transport: the soluble phase. Annu Rev Biochem.

[B10] Nakielny S, Dreyfuss G (1999). Transport of proteins and RNAs in and out of the nucleus. Cell.

[B11] Yedavalli VS, Neuveut C, Chi YH, Kleiman L, Jeang KT (2004). Requirement of DDX3 DEAD box RNA helicase for HIV-1 Rev-RRE export function. Cell.

[B12] Dayton AI (2004). Within you, without you: HIV-1 Rev and RNA export. Retrovirology.

[B13] Marques SM, Veyrune JL, Shukla RR, Kumar A (2003). Restriction of human immunodeficiency virus type 1 Rev function in murine A9 cells involves the Rev C-terminal domain. J Virol.

[B14] Duchange N, Pidoux J, Camus E, Sauvaget D (2000). Alternative splicing in the human interleukin enhancer binding factor 3 (ILF3) gene. Gene.

[B15] Saunders LR, Perkins DJ, Balachandran S, Michaels R, Ford R, Mayeda A, Barber GN (2001). Characterization of two evolutionarily conserved, alternatively spliced nuclear phosphoproteins, NFAR-1 and -2, that function in mRNA processing and interact with the double-stranded RNA-dependent protein kinase, PKR. J Biol Chem.

[B16] Kao PN, Chen L, Brock G, Ng J, Kenny J, Smith AJ, Corthesy B (1994). Cloning and expression of cyclosporin A- and FK506-sensitive nuclear factor of activated T-cells: NF45 and NF90. J Biol Chem.

[B17] Langland JO, Kao PN, Jacobs BL (1999). Nuclear factor-90 of activated T-cells: A double-stranded RNA-binding protein and substrate for the double-stranded RNA-dependent protein kinase, PKR. Biochemistry.

[B18] Liao HJ, Kobayashi R, Mathews MB (1998). Activities of adenovirus virus-associated RNAs: purification and characterization of RNA binding proteins. Proc Natl Acad Sci USA.

[B19] Shin HJ, Kim SS, Cho YH, Lee SG, Rho HM (2002). Host cell proteins binding to the encapsidation signal epsilon in hepatitis B virus RNA. Arch Virol.

[B20] Isken O, Grassmann CW, Sarisky RT, Kann M, Zhang S, Grosse F, Kao PN, Behrens SE (2003). Members of the NF90/NFAR protein group are involved in the life cycle of a positive-strand RNA virus. Embo J.

[B21] Meagher MJ, Schumacher JM, Lee K, Holdcraft RW, Edelhoff S, Disteche C, Braun RE (1999). Identification of ZFR, an ancient and highly conserved murine chromosome-associated zinc finger protein. Gene.

[B22] Krasnoselskaya-Riz I, Spruill A, Chen YW, Schuster D, Teslovich T, Baker C, Kumar A, Stephan DA (2002). Nuclear factor 90 mediates activation of the cellular antiviral expression cascade. AIDS Res Hum Retroviruses.

[B23] Parrott AM, Walsh MR, Reichman TW, Mathews MB (2005). RNA binding and phosphorylation determine the intracellular distribution of nuclear factors 90 and 110. J Mol Biol.

[B24] Shim J, Lim HR, Yates J, Karin M (2002). Nuclear export of NF90 is required for interleukin-2 mRNA stabilization. Mol Cell.

[B25] Larcher JC, Gasmi L, Viranaicken W, Edde B, Bernard R, Ginzburg I, Denoulet P (2004). Ilf3 and NF90 associate with the axonal targeting element of Tau mRNA. Faseb J.

[B26] Parker LM, Fierro-Monti I, Mathews MB (2001). Nuclear factor 90 is a substrate and regulator of the eukaryotic initiation factor 2 kinase double-stranded RNA-activated protein kinase. J Biol Chem.

[B27] Reichman TW, Muniz LC, Mathews MB (2002). The RNA binding protein nuclear factor 90 functions as both a positive and negative regulator of gene expression in mammalian cells. Mol Cell Biol.

[B28] Musacchio A (2002). How SH3 domains recognize proline. Adv Protein Chem.

[B29] Zarrinpar A, Bhattacharyya RP, Lim WA (2003). The structure and function of proline recognition domains. Sci STKE.

[B30] Cesareni G, Panni S, Nardelli G, Castagnoli L (2002). Can we infer peptide recognition specificity mediated by SH3 domains?. FEBS Lett.

[B31] Agrawal V, Kishan KV (2002). Promiscuous binding nature of SH3 domains to their target proteins. Protein Pept Lett.

[B32] Coolidge CJ, Patton JG (2000). A new double-stranded RNA-binding protein that interacts with PKR. Nucleic Acids Res.

[B33] Patel RC, Vestal DJ, Xu Z, Bandyopadhyay S, Guo W, Erme SM, Williams BR, Sen GC (1999). DRBP76, a double-stranded RNA-binding nuclear protein, is phosphorylated by the interferon-induced protein kinase, PKR. J Biol Chem.

[B34] Cullen BR (2000). Nuclear RNA export pathways. Mol Cell Biol.

[B35] Cullen BR (2003). Nuclear RNA export. J Cell Sci.

[B36] Meurs E, Chong K, Galabru J, Thomas NS, Kerr IM, Williams BR, Hovanessian AG (1990). Molecular cloning and characterization of the human double-stranded RNA-activated protein kinase induced by interferon. Cell.

[B37] Ryman KD, White LJ, Johnston RE, Klimstra WB (2002). Effects of PKR/RNase L-dependent and alternative antiviral pathways on alphavirus replication and pathogenesis. Viral Immunol.

[B38] Meyer BE, Meinkoth JL, Malim MH (1996). Nuclear transport of human immunodeficiency virus type 1, visna virus, and equine infectious anemia virus Rev proteins: identification of a family of transferable nuclear export signals. J Virol.

[B39] Farjot G, Sergeant A, Mikaelian I (1999). A new nucleoporin-like protein interacts with both HIV-1 Rev nuclear export signal and CRM-1. J Biol Chem.

[B40] Smulevitch S, Bear J, Alicea C, Rosati M, Jalah R, Zolotukhin AS, von Gegerfelt A, Michalowski D, Moroni C, Pavlakis GN, Felber BK (2006). RTE and CTE mRNA export elements synergistically increase expression of unstable, Rev-dependant HIV and SIV mRNAs. Retrovirology.

[B41] Malim MH, Bohnlein S, Hauber J, Cullen BR (1989). Functional dissection of the HIV-1 Rev trans-activator – derivation of a trans-dominant repressor of Rev function. Cell.

[B42] Kudo N, Wolff B, Sekimoto T, Schreiner EP, Yoneda Y, Yanagida M, Horinouchi S, Yoshida M (1998). Leptomycin B inhibition of signal-mediated nuclear export by direct binding to CRM1. Exp Cell Res.

[B43] Soros VB, Carvajal HV, Richard S, Cochrane AW (2001). Inhibition of human immunodeficiency virus type 1 Rev function by a dominant-negative mutant of Sam68 through sequestration of unspliced RNA at perinuclear bundles. J Virol.

[B44] Orsini MJ, Thakur AN, Andrews WW, Hammarskjold ML, Rekosh D (1995). Expression and purification of the HIV type 1 Rev protein produced in Escherichia coli and its use in the generation of monoclonal antibodies. AIDS Res Hum Retroviruses.

